# Supplementary material for: Rapid Diagnostic Test to Detect and Discriminate Infectious Hematopoietic Necrosis Virus (IHNV) Genogroups U and M to Aid Management of Pacific Northwest Salmonid Populations
Source: Animals (Basel). 2022 Jul 9;12(14):1761. doi: 10.3390/ani12141761 (PMC9311590; doi:10.3390/ani12141761)
Supplement: Supplementary file 1 [file animals-12-01761-s001.zip › animals-1756743-supplementary.pdf]

animals MDPI

SUPPLEMENTARY Figure S1: Artificial positive control (APC) and gBlock gene fragment nucleotide sequences designed for the U/M RT-rPCR assay controls. Constructs designed as previously described [26]. The construct includes an arbitrary tag sequence that can be detected by the probe described in [26].

5′ – CCA TGG TAA TAC GAC TCA CTA TAG GGC GAC AAT GGG AAC CAA GGC TAT CTA TGG GAT CAT TCT CAT TAC GTC TAG CAT CCA TGC CAC CGC CGC TAG AAG CCA TCG CTG CCA GAC AAG GCA TGT GCG CCA ACG GAG TAT TCT ATA GTG TCA CCT AAA TAC TAG T – 3′

SUPPLEMENTARY Table S1: GenBank accession numbers of the *Novirhabdovirus* nucleocapsid (N) sequences that were analyzed for the development of the U/M RT-rPCR assay. Additional in-house sequences were also examined.

Virus[1]	Strain/Genogroup	Location	Year	Genbank Accession #
IHNV	RB1/U	OR, USA	1975	U50402
IHNV	BLK94/U	WA, USA	1994	U50402
IHNV	Cdr12/U	WA, USA	2012	U50402
IHNV	RU9/U	Russia	2001	U50402
IHNV	RU1/U	Russia	2000	FJ265715
IHNV	DW10/U	ID, USA	2010	AY442508
IHNV	WRAC/M	ID, USA	1982	AY442518
IHNV	LR80/M	WA, USA	1980	AY442514
IHNV	220-90/M	ID, USA	1990	AY442514
IHNV	MC30/M	ID, USA	1991	AY442514
IHNV	Mer95/M	WA, USA	1995	AY442514
IHNV	DW09/M	ID, USA	2009	AY442514
IHNV	Qts07/M	WA, USA	2007	AY442514
IHNV	Hg508[2]/M	ID, USA	2014	ON564424
IHNV	17-073[2]/M	ID, USA	2012	ON564425
IHNV	17-054[2]	ID, USA	1999	ON564426
IHNV	32-87/E	France	1987	X89213
IHNV	20101008/J	China	2008	KJ421216
IHNV	HLJ-09/J	China	2009	JX649101
VHSV	Makah/IVa	WA, USA	1988	X59241
VHSV	MI03/IVb	MI, USA	2003	DQ427105
VHSV	DK-3592B/Ia	Denmark	1986	KC778774
HIRRV	8401H	Japan	1984	FJ376982
SHRV	n/a	Thailand	1986	AF147498

[1] Infectious hematopoietic necrosis virus (IHNV), viral hemorrhagic septicemia virus (VHSV), hirame rhabdovirus (HIRRV), and snakehead rhabdovirus (SHRV).

[2] Three IHNV strains that had variation in either the U/M or N Uni RT-rPCR primer/probe locations.
